# In vitro and pilot in vivo imaging of 18 kDa translocator protein (TSPO) in inflammatory vascular disease

**DOI:** 10.1186/s13550-021-00786-7

**Published:** 2021-05-05

**Authors:** Romain Schollhammer, Sébastien Lepreux, Nicole Barthe, Delphine Vimont, Anne Rullier, Igor Sibon, Xavier Berard, Andrea Zhang, Yasuyuki Kimura, Masahiro Fujita, Robert B. Innis, Paolo Zanotti-Fregonara, Clément Morgat

**Affiliations:** 1 Nuclear Medicine Department, University Hospital of Bordeaux, 33076 Bordeaux, France; 2 University of Bordeaux, INCIA, UMR5287, 33400 Talence, France; 3 CNRS, INCIA, UMR5287, 33400 Talence, France; 4 Pathology Unit, CH de Libourne, 33505 Libourne, France; 5 BioTis, Inserm U1026, Bordeaux, France; 6 Histologic Department, University Hospital of Bordeaux, 33076 Bordeaux, France; 7 Neurology Department, University Hospital of Bordeaux, 33076 Bordeaux, France; 8 Vascular Surgery Department, University Hospital of Bordeaux, 33076 Bordeaux, France; 9 Molecular Imaging Branch, NIMH, Bethesda, MD USA; 10 Nuclear Medicine Department, University Hospital of Bordeaux, Place Amélie Raba Léon, 33000 Bordeaux, France

**Keywords:** TSPO, PET/CT, Inflammation, Atherosclerosis, Vasculitis

## Abstract

**Background:**

Inflammatory vascular disease of the arteries, such as inflamed atheromatous plaques or arteritis, may cause aneurysms or ischemic strokes. In this context, using positron emission tomography (PET) to image inflammation may help select patients who would benefit from appropriate therapeutic interventions. This study sought to assess the usefulness of the 18 kDa translocator protein (TSPO) tracers [^11^C]-PBR28 and [^18^F]-PBR06 for imaging inflammatory vascular disease in vitro and in vivo. Immunohistochemistry for macrophage infiltration as well as autoradiography with [^18^F]-PBR06 were performed on eight paraffin-embedded, formalin-fixed atherosclerosis plaques prospectively collected after carotid endarterectomy of eight patients affected by ischemic stroke. Six different patients, one of whom was also included in the in vitro study, underwent PET imaging. Two patients with carotid stenosis associated with ischemic stroke were imaged with [^18^F]-PBR06 PET/CT, and four other patients (three with large vessel vasculitis and one with bilateral carotid stenosis but without stroke) were imaged with [^11^C]-PBR28.

**Results:**

All in vitro sections showed specific binding of [^18^F]-PBR06, which co-localized with immunohistochemistry markers for inflammation. However, in vivo TSPO imaging with either [^11^C]-PBR28 or [^18^F]-PBR06 was negative in all participants.

**Conclusion:**

Despite good uptake on surgical samples in vitro, [^11^C]-PBR28 and [^18^F]-PBR06 are not viable clinical tools for imaging inflammatory vascular disease.

*Trial registration*: NCT02513589, registered 31 July 2015 and NCT00547976, registered 23 October 2007. https://clinicaltrials.gov.

## Background

Inflammation in the vascular wall plays an important role in the pathophysiology of atherosclerosis, including the development of plaque, plaque destabilization, and rupture. In the carotid artery, a plaque may become symptomatic when it suddenly swells or promotes the formation of thrombosis and downstream embolism. As an example, stenosing atherosclerotic disease in the carotid artery is present in a large number of stroke patients [1]. In addition, large vessel vasculitis, such as Takayasu’s arteritis and Giant Cell arteritis, is characterized by a cellular immune response involving T cells and macrophages [2]. These conditions are challenging from a diagnostic perspective, because arteritis affecting the aortic arch may delay the clinical diagnosis of potentially life-threatening alterations in hemodynamic parameters.

In this context, a noninvasive method of detecting inflammatory activity, such as the accumulation of macrophages, in arteries would be of great clinical value to orient prognosis, direct therapy, and assess novel therapies. While standard imaging modalities can quantify the degree of stenosis and the thickness of an inflamed arterial wall, they cannot directly identify an inflammatory component. The positron emission tomography (PET) tracer [^18^F]-FDG, is clinically used to diagnose vascular inflammation [3, 4] but may also aspecifically accumulate in any metabolically active tissues or cells. Radiopharmaceuticals targeting 18 kDa translocator protein (TSPO), a protein heavily expressed in macrophages, may identify the presence of inflammation in the vascular wall more specifically and perhaps earlier. Indeed, recent findings underscored the interest of TSPO imaging in inflamed plaque, because TSPO co-localized with a subset of CD11b + macrophages which are recruited in advanced atherosclerotic lesions [5]. Pilot comparative studies suggest the superiority of TSPO-based radiopharmaceutical to image vulnerable plaque vs [^18^F]-FDG [6]. For instance, ex vivo autoradiography with [^3^H]-PK11195 suggests that TSPO-imaging of macrophages in atherosclerotic plaque may be feasible [7]. In humans, PET imaging with [^11^C]-(*R*)-PK11195 correlated well with [^3^H]-PK11195 binding in ex vivo samples [8]. Additionally, PET imaging with [^11^C]-(*R*)-PK11195 differentiated symptomatic from asymptomatic patients with atherosclerosis [8]. However, PK11195 has moderate affinity for TSPO and low specific binding [9]. Thus, recently developed TSPO radioligands with higher affinity for the target [10] may be more useful for in vivo imaging of vascular wall inflammation. Among these recently developed radioligands, [^18^F]-PBR06 has been successfully used in mice to image macrophage infiltration in atherosclerotic plaques, but no human imaging has been performed so far [11].

This study sought to assess the feasibility of using second generation TSPO imaging to identify vascular inflammation by performing [^18^F]-PBR06 autoradiography on surgical samples collected from unstable carotid plaques. In addition, patients with inflamed carotid plaques and large vessel vasculitis were scanned using one of two TSPO tracers: [^11^C]-PBR28 and [^18^F]-PBR06.

## Methods

### In vitro study

#### Patients

Atherosclerotic plaque samples were collected after carotid endarterectomy in eight patients affected by ischemic stroke (Table 1).

**Table 1:** Characteristics of patients in the in vitro and in vivo parts of the study

Patients	Age (years)	Sex	Stenosis localization	Stenosis degree	TSPO affinity
In vitro					
1	83	Male	Right internal carotid (IC)	70%	N/A
2	51	Male	Left IC	80%	N/A
3	87	Male	Right IC	70%	N/A
4	66	Male	Right IC	60%	High Binder
5	78	Male	Right IC	90%	Mixed Binder
6	79	Male	Left IC	50%	Low Binder
7*	81	Male	Right IC	60%	Mixed Binder
8	76	Male	Right IC	75%	Mixed Binder
In vivo					
1*	81	Male	Right IC	60%	Mixed Binder
2	57	Male	Right IC	90%	High Binder
3	78	Male	Bilateral IC	75% and 85%	N/A
4	67	Female	Bilateral Temporal Arteritis		N/A
5	76	Female	Bilateral Temporal Arteritis		N/A
6	45	Female	Bilateral carotid Left subclavian Distal aorta	Wall thickening	N/A

Asterisks identify the patient who had a PET scan after his surgical sample was analyzed in vitro. Affinity binding genotype was unknown for some participants in the in vivo part of the study because they were imaged before the different binding affinity classes were discovered.

One sample was from a participant with high-affinity binding to the TSPO protein [12], three were from mixed-affinity binders, and one was from a low-affinity binder; genotype was not determined in the remaining three participants. After embedding the samples in paraffin and fixing in formalin, four adjacent sections were used: one for hematoxylin eosin saffron (HES) staining, one for immunohistochemistry, and two for autoradiography, which were used to calculate specific and nonspecific TSPO binding, respectively.

In both the in vitro and in vivo studies, all participants gave written informed consent. Participation was approved by either the ethical committee of the University of Bordeaux, France (CNIL, declaration number 1858342v0, 11/05/2015 and CPP DC2015/01 on 12/02/2015 for the in vitro study and 2016/95, on 21/12/2016 for the in vivo study, Clinical Trial NCT02513589), where the [^18^F]-PBR06 scans were conducted, or by the Institutional Review Board (IRB) of the National Institute of Mental Health (NIMH) in Bethesda, Maryland, USA (Clinical Trial NCT00547976), where the [^11^C]-PBR28 scans were conducted.

#### Immunohistochemistry

The immunohistochemical study was performed using an automated immunostainer (Dako A/S, Glostrup, Denmark), after heat-induced retrieval in citrate buffer with antibodies against α-smooth muscle actin (α-SMA, clone 1A4, 1/100), h-caldesmon (clone h-Cd, 1/100), CD45 (clone 2B11, 1/100), CD3 (polyclonal, 1/200), and CD68 (clone PGM1, 1/100) (Dako Omnis). The epitopes were detected using the Envision+ system horseradish peroxidase detection kit and revealed with liquid diaminobenzidine (Dako Omnis).

α-SMA and h-caldesmon stains were used to quantify the percentage of the fibrous rupture of the tunica media of the muscular arteries. α-SMA stains also evaluated the infiltration of myofibroblasts in the intima. CD45, CD3, and CD68 stains assessed the count of leukocytes, T lymphocytes, and macrophages per 10 high magnification (× 400) fields, respectively.

#### Autoradiography

As previously described [13, 14], after dewaxing, rehydration, and unmasking, samples were incubated for 60 min with a binding solution containing 1 MBq/300 µL of [^18^F]-PBR06. To assess non-specific binding, a > 100-fold excess (1 μM) of cold compound [^nat^F]-PBR06 was added in adjacent slices. Samples were then rinsed for 10 min in Tris-buffered saline and distilled water and dried using air stream. Autoradiographic images were acquired over 60 min using a BetaImager-2000 (BiospaceLab, Nesles-la-Vallée, France).

After fusion by affine transformation of HES and autoradiographic images, regions of interest (ROIs) were used to calculate the amount of specific binding. A first ROI was drawn on the carotid tissue (whole plaque or macrophage compartment or smooth-muscle compartment) to estimate total binding and a second one, corresponding to background noise, was placed around the tissue. The same ROIs were then transferred to the adjacent slice to determine nonspecific binding. After subtracting background noise, specific binding was expressed as a percentage over total binding.

### In vivo study

#### Patients

Six patients were scanned (Table 1). Two patients suffering from ischemic stroke were scanned with [^18^F]-PBR06 (one of these was included in the in vitro portion of this study). Another patient with bilateral carotid stenosis, but without stroke, was scanned with [^11^C]-PBR28. In addition, three patients with large vessel vasculitis (two with Giant Cell arteritis and one with Takayasu’s arteritis) were scanned with [^11^C]-PBR28.

#### CT angiography, PET/CT acquisitions, and analysis

CT angiography was performed with a General Electric Optima 600, using Omnipaque 350 as contrast agent.

[^18^F]-PBR06 scans were performed with a Discovery RX PET/CT machine (General Electric Medical System) at the University Hospital of Bordeaux, France. Dynamic cervical PET/CT dynamic images were acquired over 90 min (27 frames composed of 6 × 30 s, 3 × 1 min, 2 × 2 min and 16 × 5 min) after intravenous administration of 166.8 ± 6.3 MBq of [^18^F]-PBR06. The images were reconstructed using an ordered-subset expectation maximization algorithm, and the CT scan was used for attenuation correction.

[^11^C]-PBR28 scans were performed with an Advance PET/CT machine (General Electric Medical System) at the NIH Clinical Center in Bethesda, Maryland, USA. After injection of 700 MBq ± 29 MBq of [^11^C]-PBR28, dynamic cervical PET/CT images were acquired over 120 min for the patient with carotid stenosis (33 frames composed of 6 × 30 s, 3 × 1 min, 2 × 2 min and 22 × 5 min), and an acquisition from the head to mid-thigh was performed for the three participants with large vessel disease (dynamic scan composed of 4 bed positions of 4 frames x 15 s, 3 × 30 s, 3 × 1 min, 3 × 2 min, and 4 × 4 min). The images were reconstructed using an ordered-subset expectation maximization algorithm, and attenuation correction was achieved with a ^68^Ge rotating pin source.

#### Statistical analysis

Mean signal for unblocked and blocked samples were compared using the Wilcoxon test for paired samples. Significance was set at *p* < 0.05 (two-tailed). Statistics were performed using GraphPad Prism software (v6.01, San Diego, CA, USA).

## Results

### In vitro study

#### Immunohistochemistry

**Fig. 1:**
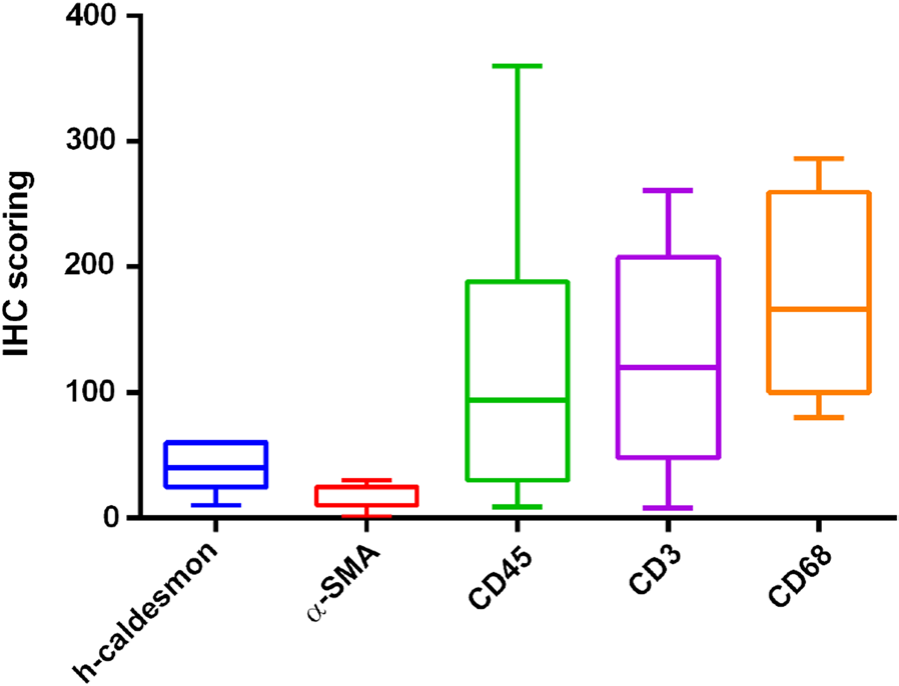
Immunohistochemistry scoring of h-caldemon, α-smooth muscle actin (α-SMA), CD45, CD3 and CD68 investigated in this study. α-SMA and h-caldesmon stains were used to quantify the percentage of the fibrous rupture of the tunica media of the muscular arteries CD45, CD3, and CD68 stains assessed the count of leukocytes, T lymphocytes, and macrophages per 10 high magnification (× 400) fields, respectively

**Fig. 2:**
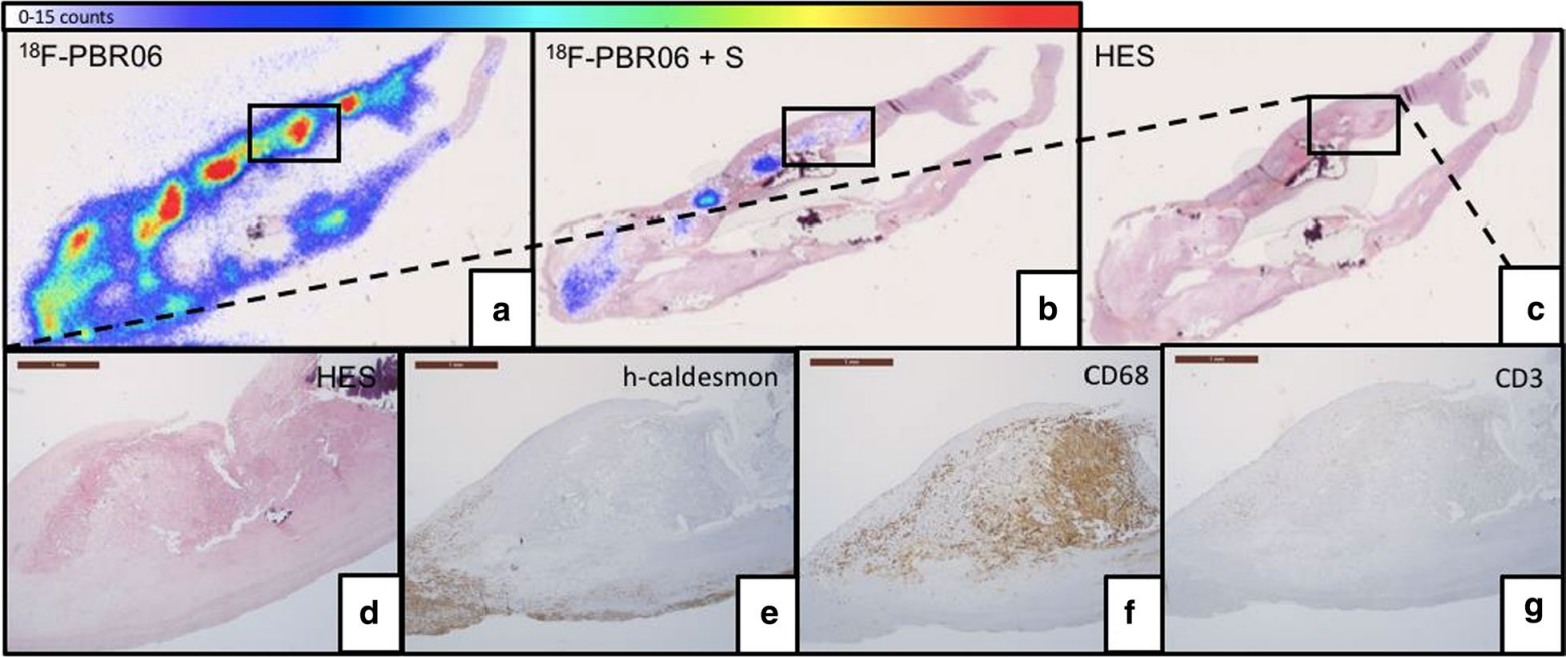
[^18^F]-PBR06 autoradiography at baseline (**a**) and after pre-incubation with cold [^nat^F]-PBR06 (**b**) and HES anatomical sections (**c**) in a piece of carotid endarterectomy. A large atheromatous plaque with calcification invaded the intima and tunica media of the arterial wall (**d**, × 25). Immunohistochemistry showed destruction of the tunica media by the plaque (**e**, × 25), macrophage infiltration around the cholesterol clefts (**f**, × 25), and scattered T-lymphocytes (**g**, × 100) within the plaque. The brown bar in the upper left corner is a 1-mm scale bar

Immunostaining consistently showed large infiltration of inflammatory cells in the artery wall. For leukocytes, nine to 360 cells were stained using CD45. For T lymphocytes, eight to 261 cells were stained using CD3. For macrophages, 82 to 286 were stained using CD68. α-SMA stained one to 30 intimal myofibroblasts and smooth muscle cells. The fibrous changes of the tunica media ranged between 10 and 60% of the wall circumference (Figs. 1 and 2).

#### Autoradiography

**Fig. 3:**
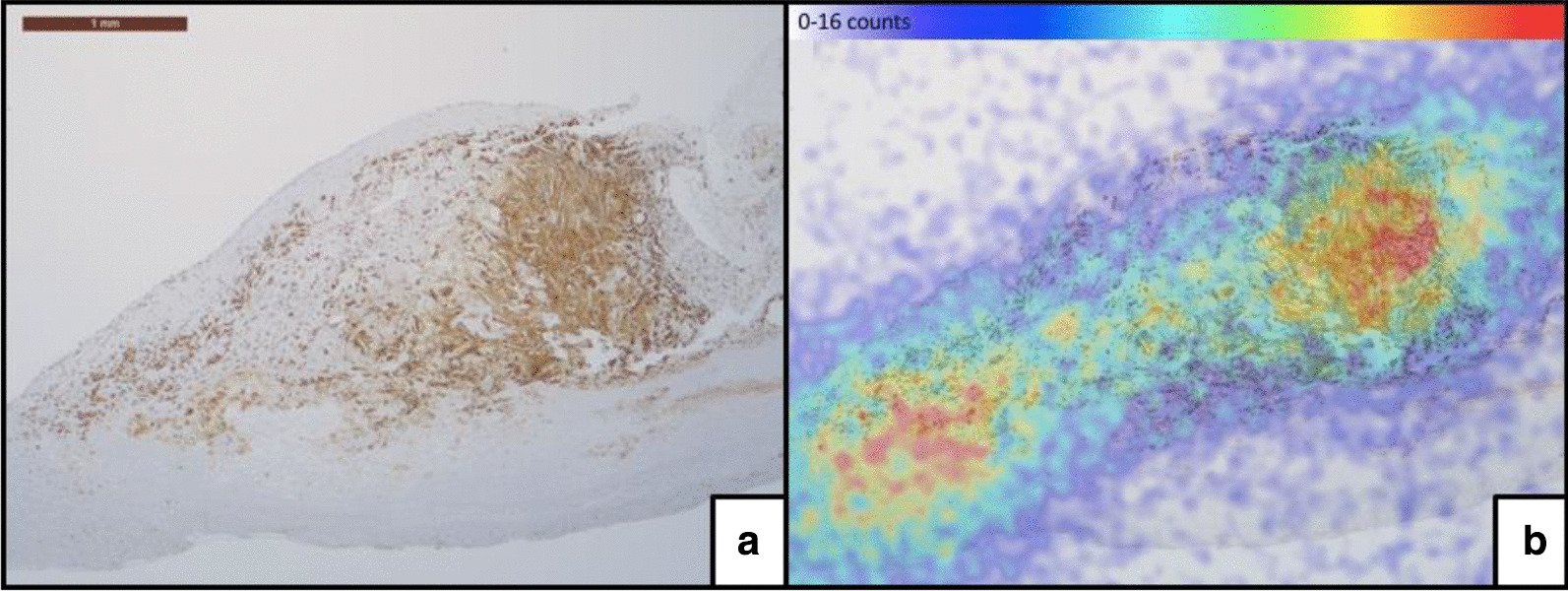
Co-localization of [^18^F]-PBR06 and CD68 macrophages. **a** CD68 immunohistochemistry. **b** fused image of [^18^F]-PBR06 micro-imaging and CD68 staining

All samples showed [^18^F]-PBR06 binding that co-localized with immunohistochemistry staining for macrophages (Figs. 2 and 3) and was displaced by the cold compound (the mean absolute count was 17.65 ± 15.01 cps/mm^2^ for total binding vs 11.04 ± 7.81 cps/mm^2^ for blocked samples, *p* = 0.0078).

**Fig. 4:**
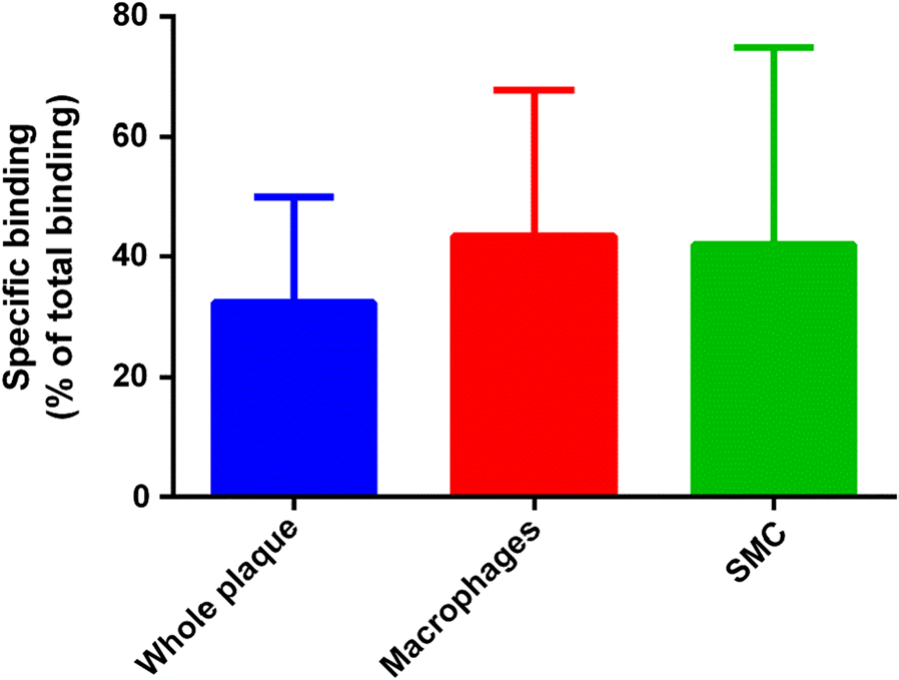
Quantification of [^18^F]-PBR06 specific binding according to various areas of atheromatous plaque (whole plaque, macrophages, and smooth muscle compartment (SMC)) of all eight samples

Specific binding was 64% of total binding in the high-affinity binding patient, 27% on average in the mixed-affinity binders, and 13% in the low-affinity binding patient. The average of all eight samples was 38 ± 20% (Fig. 4).

Interestingly, macrophages area and the smooth muscle had similar amount of specific binding.

### In vivo study

**Fig. 5:**
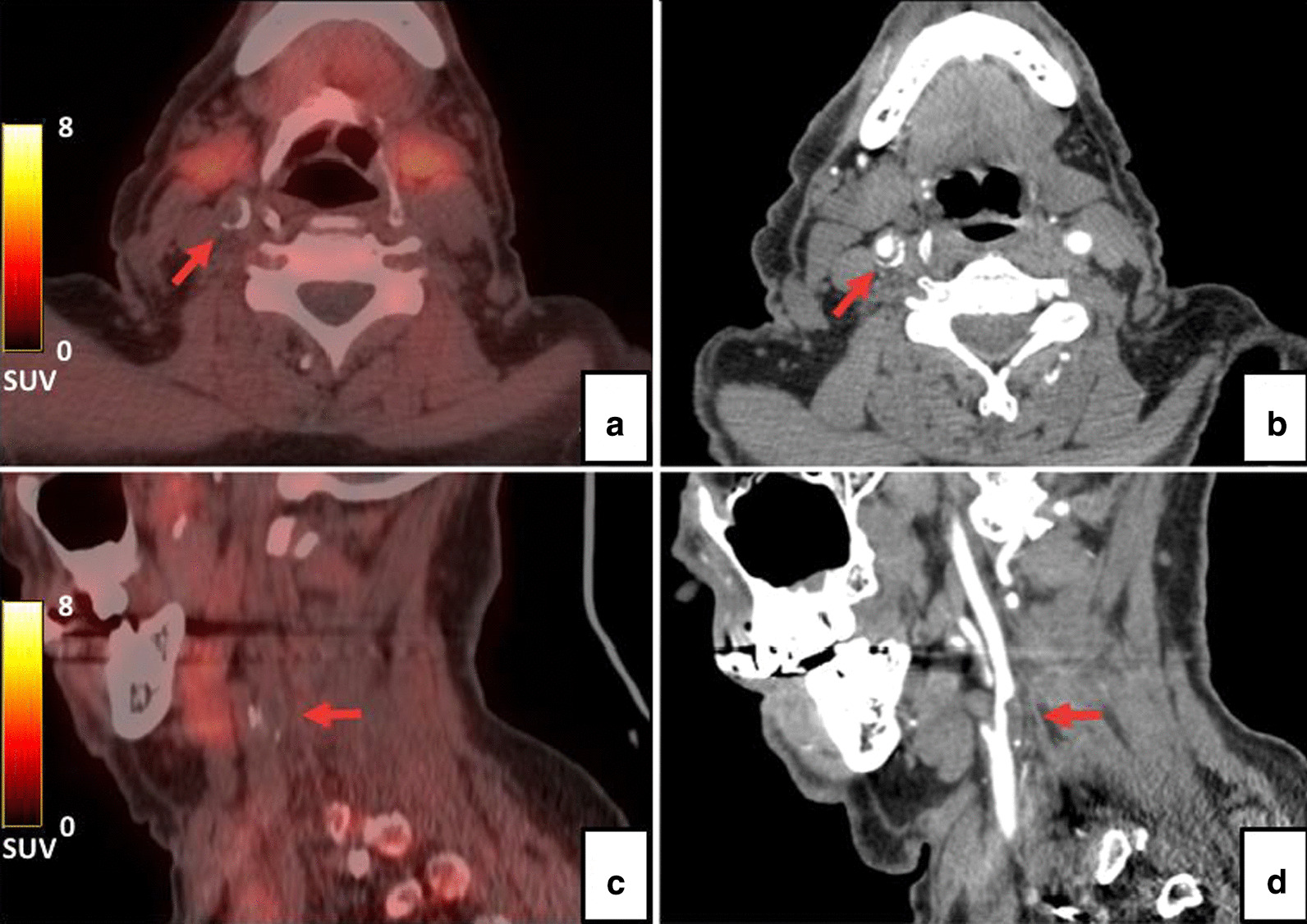
A representative [^18^F]-PBR06 PET/CT (A, C) scan and angio-CT (B, D) scan. No uptake was observed on the right carotid atheromatous plaque

PET/CT imaging was negative in all patients, regardless of whether they were scanned with [^11^C]-PBR28 or [^18^F]-PBR06 (Fig. 5).

## Discussion

This study found that [^18^F]-PBR06 showed high specific binding in vitro in surgical samples of inflamed carotid plaques. The binding level was predictably correlated to the level of affinity determined by the genotype class, but even the participant with low-affinity binding status had 13% displaceable binding. Nevertheless, PET scans for TSPO in patients detected no sign of inflammation in vivo, suggesting that TSPO imaging is not a viable clinical tool for evaluating patients with inflammatory vessel disease.

In a similar study, but limited to in vitro data, Fujimura and colleagues demonstrated that macrophage and inflammatory activity in atherosclerotic plaques could be imaged using [^3^H]-PK11195 [7]. [^18^F]-PBR06 and [^11^C]-PBR28 have equivalent imaging properties in vivo [15], and both have higher affinity for TSPO (Ki = 1.0 nM and Ki = 2.5 nM, respectively) than PK11195 (Ki = 29.2 nM). Therefore, both tracers would be more likely to show specific signal in PET studies. In this study, however, PET scans with either radioligand showed no uptake in patients with carotid stenosis or those with large vessel vasculitis. Notably, the carotid plaque of one patient (a mixed-affinity binder) was analyzed both in vitro and in vivo. While in vitro analysis showed 26% specific binding, in vivo imaging was negative.

While most radioligands bind reversibly, irreversible tracers, like [^18^F]-FDG, might have an advantage for imaging inflammation in vascular walls because their signal-to-background ratio increases over time, while the activity in the blood decreases. The signal in the carotid plaque may have been masked by the residual plasma activity and the specific uptake in blood cells because both white cells and platelets express the TSPO receptor. Indeed, as we confirmed in this study (Fig. 4), SMC specifically bind TSPO ligands [16] thus adding another challenge when using this type of imaging for stratification [17].

The limitations of this study are: first, the in vitro study concerned only patients with unstable plaques while in vivo studies were performed on patients with atheroma and large vessel vasculitis. Although these conditions have different inflammatory cells infiltrates, both are characterized by overexpression of TSPO [18]. Second, TSPO polymorphism was unknown for most of our patients (4/6) who underwent PET/CT imaging. However, the frequency of LAB genotype in the general population (9%, [12]) makes the probability of negative results due to low-binding negligible (there is 1 chance in 10,000 that the 4 subjects for whom the genotype is unknown are all low-binders). Third, direct TSPO staining was not performed, so other markers, such as CD68, were taken as a proxy for TSPO expression [8].

Taken together, the results indicate that [^11^C]-PBR28 and [^18^F]-PBR06 are not viable clinical tools for imaging inflammatory vascular diseases.

## Conclusion

Despite good uptake on surgical samples in vitro, [^11^C]-PBR28 and [^18^F]-PBR06 are not viable clinical tools for imaging inflammatory vascular disease.

## Data Availability

The datasets used and/or analyzed during the current study are available from the corresponding author on reasonable request.
